# Comparison of coronary plaque, coronary artery calcification and major adverse cardiac events in Chinese outpatients with and without type 2 diabetes

**DOI:** 10.1186/s40064-016-3373-0

**Published:** 2016-09-29

**Authors:** Lijie Zhu, Jingjing Liu, Chuanyu Gao, Wenli Zhao, Jing Que, Xianpei Wang, Datun Qi, Jun Liu, Wentao Xiao, Juanjuan Yan, Wentao Li, You Zhang, Honghui Yang

**Affiliations:** 1 Department of Cardiology, Zhengzhou University People’s Hospital, 7 Wei Wu Road, Zhengzhou, 450003 China; 2 Henan Institute of Cardiovascular Epidemiology, Zhengzhou, China; 3 Cardio-Pulmonary Function Department, Zhengzhou University People’s Hospital, 7 Wei Wu Road, Zhengzhou, 450003 China; 4 Cardiac Catheterization Room, Zhengzhou University People’s Hospital, Zhengzhou, China

**Keywords:** Type 2 diabetes, Coronary artery calcification scores, Major adverse cardiac events

## Abstract

**Objective:**

Diabetes substantially increases the risk of cardiovascular disease (CAD) and is associated with an increased risk of CAD mortality. The purpose of this study was to investigate the differences in coronary artery plaque, coronary artery calcification (CAC) measured in outpatients with and without type 2 diabetes, and the occurrence rate of a major adverse cardiac event (MACE) throughout follow-up with the same patients.

**Methods:**

Five hundred eighty-eight outpatients with suspected CAD comprising 208 diabetic and 380 non-diabetic patients were enrolled in this study. Coronary artery plaque and CAC scores were detected and measured by dual-source computed tomography. The major MACE during the follow-up period (4.0–20 months) was recorded and its relationship to type 2 diabetes and CAC was investigated.

**Results:**

The diabetes group had higher CAC scores in the left anterior descending, left circumflex, and right coronary arteries and total CAC burden than the group without diabetes. The diabetes group had more diseased coronary segments and more obstructed vessels than the non-diabetes group. Logistic regression analysis demonstrated that diabetes is positively associated with mixed coronary plaque and non-calcified plaque. All patients in the diabetes group and all patients with higher CACs in both groups had a higher incidence rate of MACEs.

**Conclusion:**

Patients with type 2 diabetes have a higher prevalence of obstructive CAD, higher CAC scores, and a higher incidence rate of MACEs than those without diabetes. Diabetes and higher CAC scores were the important predictors of the occurrence of MACEs throughout follow-up with patients.

## Background

Patients with type 2 diabetes have a two- to four-times-higher risk of cardiovascular events and cardiovascular diseases (CVDs), in which coronary artery disease (CAD) is the major cause of death, than patients without diabetes (Beckman et al. 2002; Elkeles 2010). The pathophysiology of vascular disease in diabetics involves abnormalities in endothelial and vascular smooth muscle cells (VSMCs) and alterations in platelet function (Paneni et al. 2013; Howangyin and Silvestre 2014). Vascular calcification is also more common in patients with diabetes (Abedin et al. 2004; Parhami et al. 1996; Shemesh et al. 2013; Hruska et al. 2005). Coronary artery calcification (CAC) assessed by cardiac computed tomography (CT) is a well-established surrogate marker of the total burden of atherosclerosis, a disease in which plaque deposits build up in the arteries (Rumberger et al. 1995; Sangiorgi et al. 1998; Greenland et al. 2010). CAC can predict CAD and the risk of cardiovascular events beyond the standard risk factors (Budoff et al. 2006; Detrano et al. 2008). Although the association between diabetes and CAC is controversial, several studies have indicated a significant relationship between high CAC and diabetes (Elkeles et al. 2008; Bulugahapitiya et al. 2009; Qu et al. 2003; Raggi et al. 2004); however, studies on the relationship between CAC and diabetes and major adverse cardiac events (MACEs) in Chinese patients are rare. In this study, we measured CAC in patients with and without diabetes to investigate the differences in coronary artery plaque, CAC in outpatients with and without diabetes, and the occurrence rate of MACEs during follow-up time in the same patients.

## Patients and methods

### Study population

The study protocol was approved by the Ethics Committee of the Zhengzhou University People’s Hospital. All patients were given an informed consent form that was approved by the Ethics Committee before being enrolled in this study. Six hundred fifteen consecutive Chinese outpatients with and without diabetes were surveyed. And all of the diabetes were the type 2 diabetes. Three ways to diagnose diabetes are possible before they were treated dietary intervention, oral glucose-lowing medication or insulin: (1).Symptoms of diabetes plus casual plasma glucose concentration ≥200 mg/dl (11.1 mmol/l). Casual is defined as any time of day without regard to time since last meal. The classic symptoms of diabetes include polyuria, polydipsia, and unexplained weight loss; (2).Fasting plasma glucose (FPG) ≥126 mg/dl (7.0 mmol/l). Fasting is defined as no caloric intake for at least 8 h; (3) 0.2-h plasma glucose (PG) ≥200 mg/dl (11.1 mmol/l) during an oral glucose tolerance test (OGTT) using a glucose load containing the equivalent of 75 g anhydrous glucose dissolved in water (Expert Committee on the Diagnosis and Classification of Diabetes Mellitus 2003). All patients with suspected CAD (defined when patients had symptoms such as atypical chest pain, chest tightness, shortness of breath, or an atypical change in the ST–T wave on an electrocardiography) underwent dual source computed tomography (DSCT) because of uncomfortable symptoms (chest pain, chest tightness, shortness of breath, palpitations) at Zhengzhou University People’s Hospital between June 2012 and February 2014. Of these patients, 27 (4.4 %) were lost during follow-up; therefore, baseline and follow-up data were available for only 588 patients. A complete patient history comprising present and previous illnesses was recorded and a complete physical examination was conducted.

### Study criteria

The exclusion criteria were a history of thyroid problems, history of CAD, serious cardiac arrhythmias, chronic kidney and hepatic diseases, poor CT scan quality, and a history of coronary artery stenting or bypass. And the type 1 diabetes were excluded. Known CAD was defined as a history of diagnosed myocardial infarction or the presence of coronary artery luminal narrowing >50 % documented by angiography.

### Laboratory tests

Blood samples were collected within 24 h before or after DSCT to detect the following laboratory parameters: total cholesterol, triglycerides, high-density lipoprotein (HDL-C), low-density lipoprotein (LDL-C), lipoprotein a (LPa), and cystatin C (CYSC) after the patients had fasted for at least 12 h. The reference ranges of HDL-C, LDL-C, LPa, CYSC in use in our laboratory are as follows: HDL-C = 1.2–1.68 mmol/l, LDL-C = 1.9–3.12 mmol/l, LPa = 0–30 mg/dl, CYSC = 0.55–1.05 mg/l.

### Dual-source CT angiography

DSCTs were performed using a SOMATOM Definition DSCT scanner (Siemens Medical Solutions, Forchheim, Germany). Each patient enrolled in the study was in normal sinus rhythm at the time of the DSCT scan; therefore, a beta-blocker was not needed to reduce the heart rate (Zimmerman et al. 2014). A non-enhanced scan was performed for CAC scoring. The scanning area extended from the level of the tracheal bifurcation to the diaphragm. A 70- to 90-ml bolus of iopamidol (370 mg of iodine/ml; Bracco Sine Pharmaceutical Corp. Ltd, Shanghai, China) was injected into the antecubital vein at a flow rate of 5.0 ml/s. Slices were acquired under the following conditions: tube voltage 100–120 kV (adapted to body mass index [BMI]); tube current, 220 mA; collimation, 64 × 0.6 mm, slice collimation 2.0 × 64 × 0.6 mm by means of a z-flying focal spot; rotation time, 0.33 s; and pitch, 0.2–0.5 depending on heart rate.

### Image analysis

DSCTA images were interpreted by two experienced cardiovascular radiologists blinded to all patient characteristics. CAC was quantified using Syngo CaScore (Siemens Medical Solutions, Forchheim, Germany) and the scores were measured by a CT technologist using the standard Agatston calcium scoring algorithm (Agatston et al. 1990).

### Coronary plaque assessment

According to American Heart Association standards, coronary arteries were divided into 15 segments anatomically (Austen et al. 1975). In this study, we divided the coronary arteries into the following four branches: left main (LM), left anterior descending (LAD), left circumflex (LCX), and right (RCA). We evaluated the number of diseased coronary vessels and segments of each branch and total coronary artery, number, and types of diseased segments, and the number of obstructive or non-obstructive segments. Plaques are classified as (A) calcified plaque: higher CT density than contrast-enhanced lumen; (B) non-calcified plaque: without any calcified portions (≦130 HU). (C) mixed plaques: non-calcified and calcified component in single plaque. The differences in the distribution of the three plaque subtypes were compared between patients with and without diabetes. DSCT on each patients was also scored on a per-segment basis for plaque stenosis severity. A stenosis of ≥50 % was considered obstructive CAD and the segment was considered to be an obstructive segment. The number of segments that contained all three plaque subtypes was calculated for each patients. Per coronary segment was assigned by one coronary plaque type. They were classified as mixed plaque when both mixed (or calcified) and non-calcified plaques were seen in one segment.

### Patient follow-up

Follow-up information was obtained from patient records in outpatient clinics and from telephone interviews with the patients or their immediate relatives. All interviews were conducted by the same cardiologist and he was blinded for status diabetes. The primary endpoint at follow-up was the incidence of MACEs, including death from any cause, such as nonfatal myocardial infarction (MI); percutaneous coronary intervention; and coronary artery bypass graft. Nonfatal MI was defined as the presence of typical chest pain, electrocardiographic elevation of the ST segment with or without Q waves, and serum cardiac enzyme elevation at least twice that of the normal upper limit.

### Statistical analyses

All statistical analyses were performed using SPSS version 17.0 (SPSS. Inc., Chicago, IL, USA). Continuous variables are expressed as the mean ± standard deviation and categorical variables are described using number and percentage. The Student’s *t* test was used to compare continuously normal variables and the Mann–Whitney U test was used for continuously abnormal variables; a Chi squared test was used for categorical variables. The difference in the presence of the three subtype plaque segments were compared using cox regression model to adjust for diabetes, sex, age, BMI, LDL levels, HDL levels, and LPa. The cumulative event rate was evaluated using a Kaplan–Meier estimate and compared using a log-rank test. P < 0.05 was considered to be statistically significant.

## Results

After screening, 588 patients (comprising 372 men) average aged 61.06 ± 11.89 years and of whom 208 had diabetes were followed for 14.33 ± 2.59 months. Clinical and biochemical characteristics are shown in Table 1. The diabetes group (n = 208) had significantly higher BMI (p = 0.001), LPa (p = 0.044), and CYSC levels (p = 0.002); however, this group had fewer men (p = 0.016) and lower HDL-C levels (p < 0.001) than the group without diabetes. No significant difference was observed between the two study groups for age; hypertension; TG, CHOL, LDL-C, apolipoprotein A-I (APOA1), APOA100, and homocysteine levels; and the proportion of patients with a history of smoking. In this study, parts of patients of both diabetes and non-diabetes groups had the history of stains use, although the cholesterol and APO measurements might be influenced by stains use, no significant difference was observed between the two study groups for stains use but glucose-lowering drugs.

**Table 1:** Clinical characteristics in diabetes and non-diabetes groups

Variables	Total (N = 588)	Diabetes (N = 208)	Non-diabetes (N = 380)	p
Age (years)	61.06 ± 11.89	61.91 ± 10.57	60.60 ± 12.55	0.179
Sex, male (%)	372 (63.3)	118 (56.7)	254 (66.8)	0.016
Hypertension [n (%)]	380 (64.6)	141 (67.8)	239 (62.9)	0.242
Smoking [n (%)]	264 (44.9)	86 (41.3)	178 (46.8)	0.225
BMI (Kg/m^2^)	25.54 ± 3.480	26.11 ± 2.668	25.23 ± 3.821	0.001
CHOL (mmol/l)	4.70 ± 0.934	4.78 ± 0.967	4.66 ± 0.915	0.170
TG (mmol/l)	1.70 ± 1.309	1.79 ± 1.168	1.65 ± 1.379	0.232
HDL-C (mmol/l)	1.19 ± 0.320	1.12 ± 0.304	1.22 ± 0.323	<0.001
LDL-C (mmol/l)	2.67 ± 0.691	2.74 ± 0.737	2.63 ± 0.663	0.078
APOA1 (g/l)	1.15 ± 0.229	1.14 ± 0.224	1.16 ± 0.232	0.458
APOA100 (g/l)	0.95 ± 0.261	0.96 ± 0.227	0.95 ± 0.277	0.463
LPa (mg/dl)	21.97 ± 17.800	23.97 ± 17.910	20.88 ± 17.668	0.044
HCY (µmol/l)	17.89 ± 8.533	17.12 ± 6.577	18.31 ± 9.416	0.074
CYSC (mg/l)	0.97 ± 0.282	1.02 ± 0.340	0.94 ± 0.240	0.002

Data are expressed as the mean ± SD and number (percentage)

*BMI* body mass index, *CHOL* cholesterol, *TG* triglycerides, *HDL*-*C* high-density lipoprotein cholesterol, *LDL*-*C* low-density lipoprotein cholesterol, *APO* apolipoprotein, *LPa* lipoprotein a, *HCY* homocysteine, *T score* total coronary artery calcification scores, *MI* myocardial infarction

Significant differences in CAC levels between patients with and without diabetes were observed (Table 2). The diabetes group had significantly higher CAC scores in LAD (155.03 ± 256.47 vs. 92.78 ± 195.72, respectively; p = 0.003), LCX (39.11 ± 67.74 vs. 22.97 ± 52.90, respectively; p = 0.003), RCA (88.82 ± 172.39 vs. 32.38 ± 70.40, respectively; p < 0.001), and total CAC scores (309.00 ± 457.14 vs. 180.09 ± 290.62, respectively; p < 0.001) than that in the non-diabetes group, but no differences were found in LM CAC levels between the two groups.

**Table 2:** Comparison of coronary artery calcification between diabetes and non-diabetes

Variables	Total (N = 588)	Diabetes (N = 208)	Non-diabetes (N = 380)	p
LM CAC scores	29.22 ± 116.71	22.44 ± 63.59	32.94 ± 137.30	0.207
LAD CAC scores	114.95 ± 221.10	155.03 ± 256.47	92.78 ± 195.72	0.003
LCX CAC scores	28.68 ± 59.03	39.11 ± 67.74	22.97 ± 52.90	0.003
RCA CAC scores	52.34 ± 120.04	88.82 ± 172.39	32.38 ± 70.40	<0.001
Total CAC scores	225.69 ± 363.36	309.00 ± 457.14	180.09 ± 290.62	<0.001

*LM* left main, *LAD* left anterior descending, *LCX* left circumflex, *RCA* right coronary artery, *CAC* coronary artery calcification

The diabetes group had a significantly higher number of mixed plaque segments (2.72 ± 2.45 vs. 1.69 ± 2.12, respectively; p < 0.001), non-calcified plaque segments (0.87 ± 1.36 vs. 0.49 ± 0.97, respectively; p < 0.001), and total plaque segments (4.76 ± 3.10 vs. 3.36 ± 2.58, respectively; p < 0.001) than the non-diabetes group, but no differences were observed in levels of calcified plaque between the two groups (Table 3). There were a significantly higher number of obstructed vessels (1.00 ± 1.02 vs. 0.54 ± 0.81, respectively; p < 0.001) and obstructed segments (1.92 ± 2.24 vs. 0.77 ± 1.34, respectively; p < 0.001) in the diabetes group than in the non-diabetes group.

**Table 3:** Comparison of coronary artery plaque between diabetes and non-diabetes patients

Variables	Total (N = 588)	Diabetes (N = 208)	Non-diabetes (N = 380)	p
Number of mixed plaque segments	2.05 ± 2.29	2.72 ± 2.45	1.69 ± 2.12	<0.001
Number of calcified plaque segments	1.18 ± 1.67	1.18 ± 1.70	1.18 ± 1.66	0.991
Number of non-calcified plaque segments	0.63 ± 1.14	0.87 ± 1.36	0.49 ± 0.97	<0.001
Total plaque segments	3.85 ± 2.85	4.76 ± 3.10	3.36 ± 2.58	<0.001
Number of obstructive vessels	0.70 ± 0.92	1.00 ± 1.02	0.54 ± 0.81	<0.001
Number of obstructive segments	1.18 ± 1.80	1.92 ± 2.24	0.77 ± 1.34	<0.001

Mixed plaques were significantly associated with age [odds ratio (OR) 1.029, 95 % confidence level (CI) 1.014–1.044, p < 0.001], diabetes (OR 1.796, 95 % CI 1.246–2.589, p = 0.002), and LDL-C (OR 2.041, 95 % CI 1.567–2.657, p = 0.002) (Table 4). Non-calcified plaques were significantly associated with diabetes (OR 1.574, 95 % CI 1.101–2.250, p = 0.013), and calcified plaques were significantly associated with age (OR 1.071, 95 % CI 1.054 to −1.088, p < 0.001) and BMI (OR 1.12, 95 % CI 1.029–1.141, p = 0.002).

**Table 4:** Predictors of coronary plaque by Cox regression model

Plaques	Variables	OR	95 % CI	p
Mixed plaque	Age	1.029	1.014–1.044	<0.001
	Diabetes	1.796	1.246–2.589	0.002
	LDL-C	2.041	1.567–2.657	<0.001
Non-calcified plaque	Diabetes	1.574	1.101–2.250	0.013
Calcified plaque	Age	1.071	1.054–1.088	<0.001
	BMI	1.12	1.029–1.141	0.002

*OR* odds ratio, *CI* confidence interval, *LDL*-*C* low-density lipoprotein cholesterol, *BMI* body mass index

According to the multivariate analyses, diabetes [hazard ratio (HR) = 1.136–3.753, p = 0.017] and CAC levels (HR = 1.000–1.002, p = 0.002) were independent predictors of cumulative MACEs (Table 5).

**Table 5:** Multivariate analysis of predictors of incidence rate of major adverse cardiac event

Variables	HR	95 % CI	p
Diabetes	2.065	1.136–3.753	0.017
CAC scores	1.001	1.000–1.002	0.002

*CAC* coronary artery calcification, *HR* hazard ratio, *CI* confidence interval

**Fig. 1:**
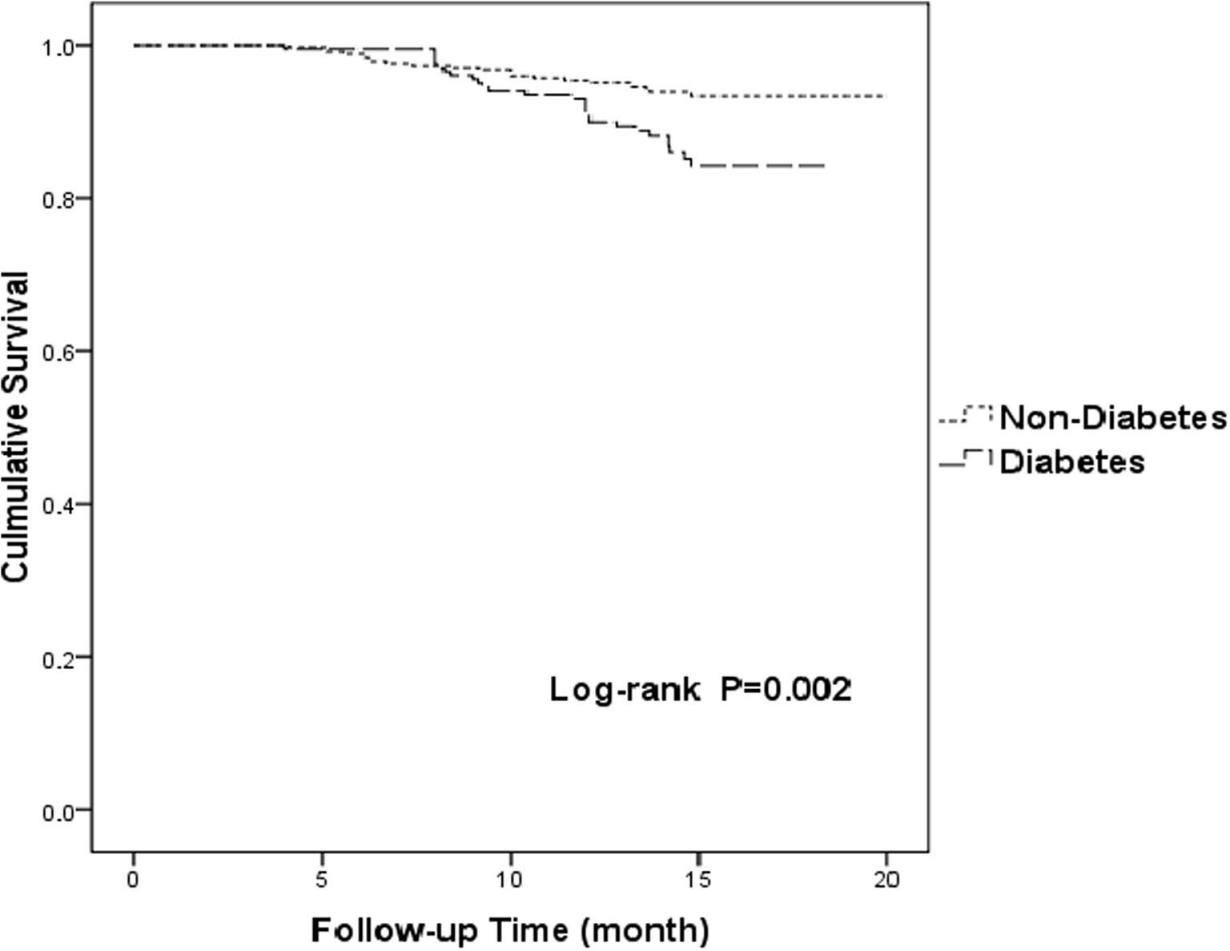
Cumulative major adverse cardiac event–free survival curves between diabetes and non-diabetes groups. *MACE* major adverse cardiac event

**Fig. 2:**
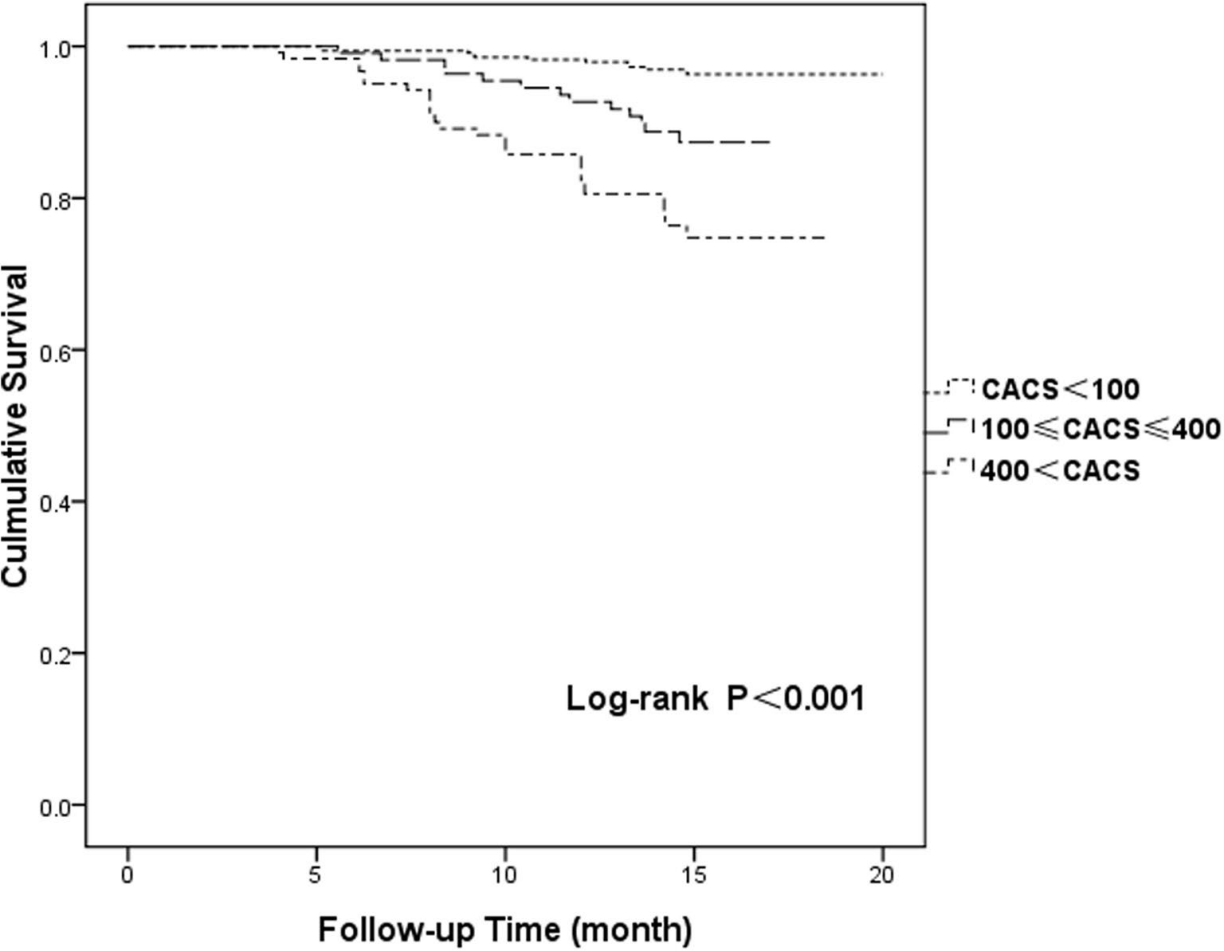
Cumulative major adverse cardiac events–free survival curves among patients with coronary artery calcium scores (CAC scores) < 100, 100 ≤ CAC scores ≤ 400 and 400 < CAC scores. *MACE* major adverse cardiac event

**Fig. 3:**
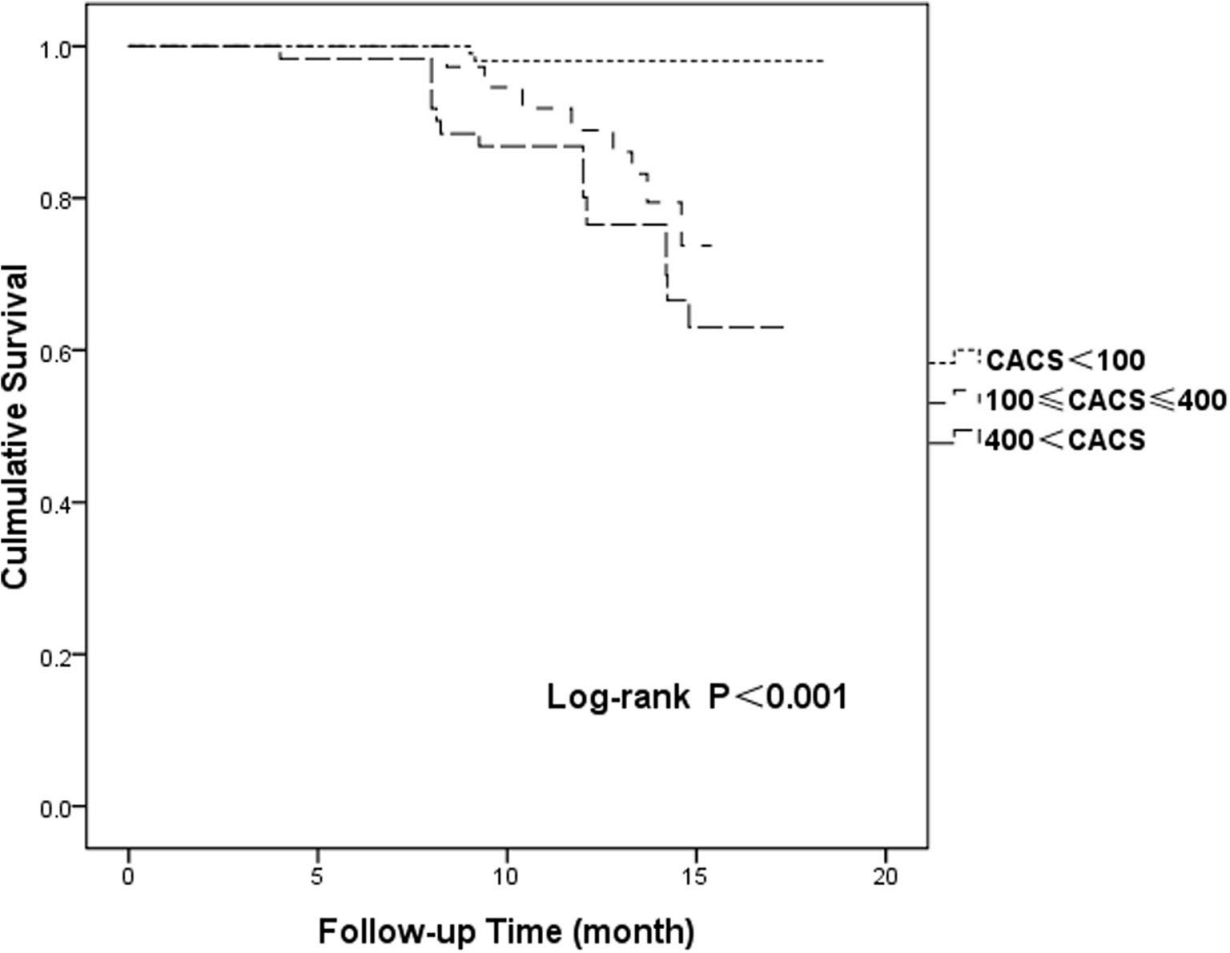
Cumulative major adverse cardiac event (MACE)–free survival curves among patients with coronary artery calcium scores (CAC scores) < 100, 100 ≤ CAC scores ≤ 400, and 400 < CAC scores in the diabetes group. *MACE* major adverse cardiac event

**Fig. 4:**
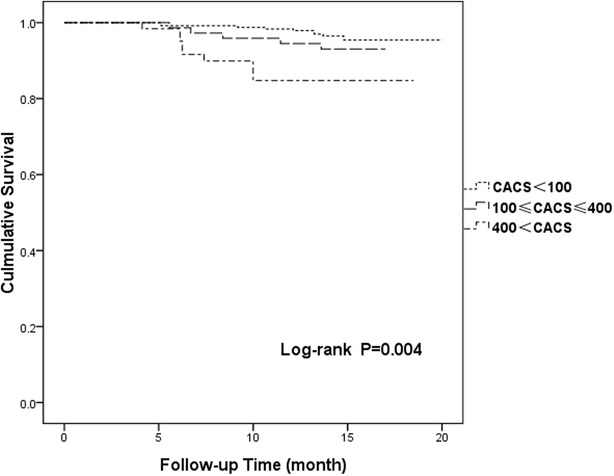
Cumulative major adverse cardiac event (MACE)–free survival curves among patients with coronary artery calcium scores (CAC scores) < 100, 100 ≤ CAC scores ≤ 400, and 400, CAC scores in the non-diabetes group. *MACE* major adverse cardiac event

Kaplan–Meier survival curves for the incidence of MACEs in patients with diabetes versus those without diabetes are shown in Fig. 1 (p = 0.002). Cumulative MACE-free survival curves among groups with CAC scores < 100, 100 ≤ C CAC scores ≤ 400, and 400 < CAC scores are shown in Fig. 2 (p < 0.001). The same relationships among groups with CAC scores < 100, 100 ≤ CAC scores ≤ 400, and 400 < CAC scores in the diabetes group are shown in Fig. 3 (p < 0.001) and those in the non-diabetes group are showed in Fig. 4 (p = 0.004).

## Discussion

First, the present study demonstrated that diabetes is related to higher levels of CAC in outpatients with suspected CAD. Second, diabetes is related to mixed plaque, non-calcified plaque, and the extent of coronary artery stenosis. After an adjustment for conventional risk factors, the relationship remained significant. Third, diabetes and CAC levels were significantly related to the incidence rate of MACEs in our patients. The results of this study suggested that diabetes was positively related to the extent of CAC and coronary artery stenosis, and both diabetes and CAC were positively related to the incidence rate of MACEs in Chinese outpatients with suspected CAD.

Several studies have shown that CAD is a leading cause of morbidity and mortality among patients with diabetes (Scholte et al. 2008; Haffner et al. 1998). Compared with people without diabetes, people with diabetes have nearly double the risk of cardiovascular disease, even after adjusting for established cardiovascular risk factors (Seshasai et al. 2011). The pathophysiology of vascular disease in patients with diabetes involves abnormalities in endothelial cells and VSMCs, and alterations in platelet function (Paneni et al. 2013; Howangyin and Silvestre 2014). CAC scores are not only a sign of atherosclerosis used to predict the likelihood of significant CAD and myocardial ischemia, but also an indicator of the total coronary atherosclerotic plaque burden (Rumberger et al. 1995). Vascular calcification is more common in patients with diabetes, which is an active process associated with complex genetic, cellular, and molecular pathways, and the disease in patients with diabetes might be related to biochemical changes (Snell-Bergeon et al. 2013; Boström et al. 2011). Chen et al. (2006) and Zhang et al. (2008) found that high serum glucose levels are associated with an increased expression of Cbfal and BMP-2 and enhanced the calcification of VSMCs. Increased levels of BMP-2 exert proinflammatory and proatherogenic effects by inducing oxidative stress and endothelial dysfunction, and have been shown to promote plaque calcification by inducing an osteogenic phenotype in VSMCs (Willette et al. 1999; Li et al. 2008). In this study, we found a similar conclusion that higher CAC scores in outpatients with suspected CAD are related to diabetes, and that oxidative stress and endothelial dysfunction induced by BMP-2 might be the important intermediate links.

In this study, we found that diabetes is related to mixed plaque, non-calcified plaque, and the extent of coronary artery stenosis. One study on diabetes and coronary artery plaques showed that non-calcified plaques are the main subtype in asymptomatic diabetes patients compared with those without diabetes (Scholte et al. 2008), which agrees with our research. In several studies, intravascular ultrasound and magnetic resonance imaging were used to detect the composition of plaque (Schartl et al. 2001; Hong et al. 2010; Qian et al. 2009; Koops et al. 2007), and the mixed plaques detected by coronary computed tomography angiography (CCTA) were associated with high-risk features (Pundziute et al. 2008). Thin-cap fibroatheromas occurred more frequently in mixed plaques than in non-calcified and calcified plaques. A recent study demonstrated that patients with diabetes had an increased number of obstructive and mixed plaque segments compared to patients without diabetes (Pundziute et al. 2008). Our study is in agreement with all of the cited studies that showed that there is a higher plaque burden and extent of atherosclerosis in patients with diabetes compared to those without diabetes. We found that there is a relatively more non-calcified plaque burden in patients with diabetes than in those without diabetes. Several studies have also confirmed the effect of diabetes on vulnerable plaque and acute coronary syndrome (ACS) (Stamler et al. 1993; Leschka et al. 2008). Unstable non-calcified plaque, was vulnerable and frequently detected in patients with ACS. We did not find any differences in the number of calcified plaque segments between the two groups, but the scores of coronary calcium were significantly different. These results might be because the volume and quality of some calcified plaques were greater in patients with diabetes than in those without diabetes, but this remains to be determined.

We discovered that diabetes and CAC are significantly related to the incidence rate of MACEs in our patients. Diabetes and high CAC scores might increase the incidence of short-term and long-term major adverse cardiac events through some of the pathways mentioned above. Diabetes is a predictive factor for MACEs, and we found that high CAC scores are a predictive factor for MACEs in the groups with and without diabetes. These results are similar to those of other studies on the effect of diabetes on MACEs during the following-up time.

### Study limitations

This study had several limitations. The study population comprised outpatients and, consequently, complete data on some parameters were not collected. As a result, it is possible that other factors that were potentially associated with CAC were not identified. This study was based on a single center. Larger studies that comprise a more diverse population would be needed to verify these findings. In this study, the prognostic value of CCTA in diabetes and non-diabetes patients was not determined, and more studies are needed to determine this value in this group of patients.

## Conclusion

Patients with diabetes have a higher prevalence of obstructive CAD, higher CAC scores, and a higher incidence rate of MACEs than those without diabetes. Diabetes and higher CAC scores are the important predictors of the occurrence of MACEs during follow-up.

## Abbreviations

CAD: cardiovascular disease
CAC: coronary artery calcification
MACE: major adverse cardiac event
VSMCs: vascular smooth muscle cells
DSCT: dual source computed tomography
HDL-C: high-density lipoprotein
LDL-C: low-density lipoprotein
LPa: lipoprotein a
CYSC: cystatin C
BMI: body mass index
LM: left main
LAD: left anterior descending
LCX: left circumflex
RCA: right coronary artery
MI: myocardial infarction
OR: odds ratio
CI: confidence level
HR: hazard ratio
CCTA: coronary computed tomography angiography
ACS: acute coronary syndrome
